# Persistent and mobile organic compounds—an environmental challenge

**DOI:** 10.1007/s00216-020-02542-7

**Published:** 2020-03-09

**Authors:** Thomas P. Knepper, Thorsten Reemtsma, Torsten C. Schmidt

**Affiliations:** 1 Europe University of Applied Sciences, Limburger Straße 2, 65510 Idstein, Germany; 2 Department of Analytical Chemistry, Helmholtz-Centre for Environmental Research - UFZ, Permoserstrasse 15, 04318 Leipzig, Germany; 3 Instrumental Analytical Chemistry and Centre for Water and Environmental Research, Universitätsstr. 5, 45141 Essen, Germany

It is commonplace in analytical chemistry that the unknown is difficult to analyse—and that it is difficult to learn about the unknown without the ability to analyse it.

To some extent, the development of non-target screening (NTS) methods in liquid chromatography-mass spectrometry (LC-MS) in the past decade seemed to overrule this experience for the field of environmental analysis. And a term like “digital sample freezing” is evidence of this understanding that NTS would provide (almost) complete information on the constituents of an environmental sample.

But analytical chemists are well aware that this is a misconception:

1. Because ionization is a prerequisite for mass spectrometric detection, only analytes are visible by LC-MS that are suitable for ionization (or the ionization suitable for the analytes).
2. Also chromatographic retention is required to separate the analytes of interest from the sample matrix that may, otherwise, disturb their ionization.

Bearing in mind that the typical NTS approach by LC-MS involves reversed phase columns, it is obvious that very polar analytes are not adequately covered—they are not retained in RPLC, coelute with much of the sample matrix, are not reliably ionized and, thus, not detected. Also‚ extraction/enrichment of very polar analytes from aqueous samples is challenging. Therefore, we have to rely on specific, often, single-compound methods to detect very polar, often ionic‚ compounds.

This “analytical gap” for very polar analytes is well documented for per- and polyfluoroalkyl substances (PFAS), where traditional methods based on RPLC-MS were applicable down to C4 compounds. But what about C3–C1? Only recently, trifluoroacetic acid and trifluoromethane sulfonic acid were shown to be widely present in an aqueous environment—with methods specifically developed for these very polar contaminants. You are invited to read more about this in this topical collection.

PFAS are not the only family with very polar members. Other examples are widely discussed contaminants‚ such as glyphosate and EDTA, but many of the highly polar contaminants are yet unknown. See the “commonplace” above.

Unfortunately, substances of very high polarity are not only a challenge for environmental analytical chemistry. If they are persistent, they also challenge the water supply because neither sorption nor chemical or biodegradation removes them efficiently in natural systems and water treatment. For such persistent and mobile organic compounds (PMOCs), there is a “protection gap”.

This combination of an “analytical gap” and a “protection gap” may be very critical as we may not recognize that a PMOC has passed through these gaps.

This topical collection in *Analytical and Bioanalytical Chemistry* provides an excellent overview of the most recent activities to close the analytical gap. It covers chromatographic methods such as HILIC, CE, SFC, IC, and MMC in combination with mass spectrometry and explores the potential of these methods‚ in general‚ or for the analysis of specific but important classes of PMOCs.

We hope that the readers of *Analytical and Bioanalytical Chemistry* will find this collection inspiring and beneficial and that it, thus, contributes to the advancement of analytical methods for highly polar compounds and to narrow down the “analytical gap”.

We thank all authors for submitting their interesting contributions for this special issue, the reviewers for their critical but constructive work, and the editorial team of *Analytical and Bioanalytical Chemistry* for its highly professional collaboration and encouragement.

